# Flexible control of an ultrastable levitated orbital micro-gyroscope through orbital-translational coupling

**DOI:** 10.1515/nanoph-2022-0625

**Published:** 2023-02-28

**Authors:** Wenqiang Li, Xia Wang, Jiaming Liu, Shuai Li, Nan Li, Huizhu Hu

**Affiliations:** College of Optical Science and Engineering, Zhejiang University, Hangzhou 310027, China; Quantum Sensing Center, Zhejiang Lab, Hangzhou 310000, China; State Key Laboratory of Modern Optical Instrumentation, Zhejiang University, Hangzhou 310027, China

**Keywords:** laser trapping, optical manipulation, optical tweezers

## Abstract

Introducing rotational degree of control into conventional optical tweezers promises unprecedented possibilities in physics, optical manipulation, and life science. However, previous rotational schemes have largely relied upon the intrinsic properties of microsphere anisotropy—such as birefringence or amorphous shape—which involves sophisticated fabrication processes and is limited in their application range. In this study, we demonstrated the first experimental realization of orbiting a homogeneous microsphere by exploiting angular momentum in a transversely rotating optical trap. The high level of rotational control allows us to explore orbital-translational coupling and realize an ultra-stable micro-gyroscope of considerable value. The dynamics of orbital levitated particle was theoretically characterized using a simple model. Our proposed method provided a novel way to qualitatively characterize optical trap features. In the future, the approach could pave the way for investigating rotational opto-mechanics, rotational ground state cooling, and the study of ultra-sensitive angular measurement.

## 1 Introduction

Delicate control and coupling over all degrees of freedom in mesoscopic regime is an issue of fundamental interest in physics [1–4]. On these length scales, optically levitated nano- or micro-particle offers an ideal platform for such goals, owing to its natural harmonic trapping potential and decoherence from the surrounding environment [5–7]. Recently, the ability to track both the center-of-mass (COM) motion and orientation of such particles has opened the rapidly growing field in rotational opto-mechanics [8–11], providing breakthroughs in fields such as quantum entanglement [12–14], micromachines [15], metrology [16, 17], and rotational-translational coupling [18–20]. However, most rotational methods exploit sphere anisotropy—such as birefringence or amorphous shape—to transfer the angular momentum of trapping beam into a spinning rotation degree of freedom [21]. This usually requires sophisticated fabrication processes [22–25], restricting the applicable range and limiting its rotational degree of control. Indeed, although rotation-induced translational cooling effect has been previously observed [11], the underlying principles of the coupling between spinning and translation remain obscure [26].

The above limitations can be overcome by optically orbiting the levitated oscillator such that flexible control over its orbiting degree is accessible. Previously, orbiting a levitated particle has been proven through the higher order of orbital angular momentum (OAM), misaligned dual-beam optical trap, and nonlinear optical effect [27–30]. Gaining control over the rotational degree of freedom is beneficial in at least two ways:

– First, it provides new insights into the orbital-translational coupling system [31]. Since the field of opto-mechanics has moved into quantum era [32, 33], the introduction of orbital degree of freedom offers new insights in fascinating fields such as macroscopic quantum entanglement or quantum friction [34, 35].
– Second, precise control over the frequency makes it possible to generate an ultrastable gyroscope of considerable value. Micro-rotors with a record high quality factor (Q) and small orbiting radius can be achieved in such a micro-gyroscope, which could be used to conduct angular measurement and study nonclassical correlations at nanoscale [36, 37].

In this study, we experimentally validated a levitated orbital rotor system in a transversely rotating optical trap. Notably, this method could be applied to all trappable levitated objects as well as provide flexible manipulation over the orbiting degree. Indeed, an exquisite control of an orbiting levitated microsphere is achievable at frequency ranging from sub-Hz to kHz and controllable orbital radius ranging from the nano- to sub-millimeter scale. The coupling of orbital and translational degree of freedom was also observed in experiments and analyzed using the Langevin model. Intuitively, the maximum couple effect could be observed when orbiting frequency coincided with the harmonic oscillation frequency. Moreover, such a process became increasingly amplified with less damping from the environment, a maximum of ten times magnification of the orbital state was observed at 1 mbar. Such a process was exploited to generate an ultrastable orbital rotor with record-high quality factor of up to 10^8^. At higher orbital frequencies, the coupling efficiency was retained and such phenomenon was exploited to realize a record small orbital radius of 10 nm. Our results demonstrated flexible control over the orbital degree of freedom in a levitated system, opening new possibility in the field of optomechanical rotation quantum coupling and metrology.

## 2 Methods

### 2.1 Experimental setup

**Figure 1:**
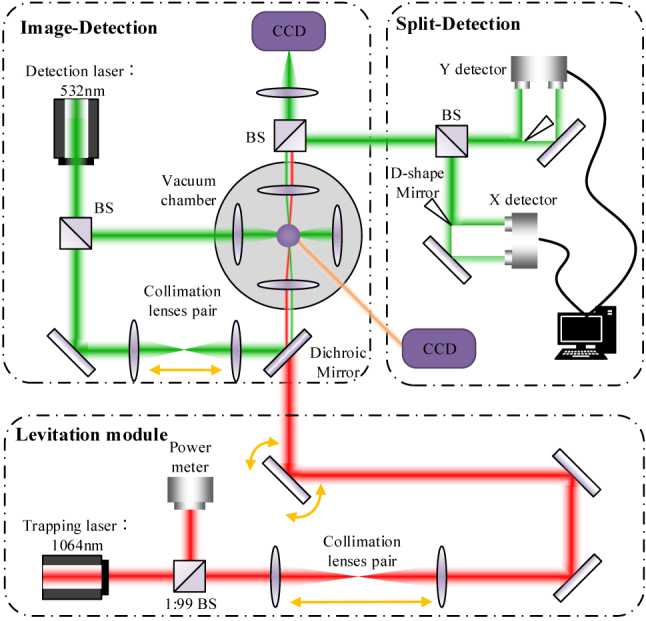
The optical setup used for the trapping and orbiting of a levitated particle in vacuum chamber.

A silica microsphere of 25 μm diameter was trapped using optical trap of 1064 nm wavelength, as shown in Figure 1. The beam was weakly focused using a lens with a nominal NA of 0.03, corresponding to a beam waist of approximately 11 μm and a focal length of 100 mm. As such, a large harmonic potential volume in three dimensions could be assured. Such a large volume trap and long focal length allow trapped sphere to orbit in a larger circle. In our case, the trapped microsphere had a diameter of 25 μm, corresponding to a mass of approximately 21.0 ng. The trapping position was set in a vacuum chamber to minimize air disturbance and analyze orbital dynamics under low vacuum. To introduce an orbiting degree of freedom for the levitated particle, a steering mirror (PSM2, Newport) was used to tilt the propagation angle of the incident trapping beam. Then the incident beam was focused to rotate the optical trap in the *x*–*y* plane, driving the trapped particle to do orbital motion.

### 2.2 Detection scheme

To obtain position of the orbital particle, we implemented both image-detection and split-detection approaches. Considering that the trapping beam is orbiting while driving the sphere, an extra beam of 532 nm wavelength is used to track the trajectory position of the sphere. An aspherical lens was used to collect scattering light, which was later split by a non-polarization BS (beam splitter). In image-detection scheme, an illumination area around the sphere was generated by adjusting the collimation lens pair. The levitation and orbiting of the particle could be directly observed by a CCD camera at 25 frames/s.

Although image-detection offers real-time video about the motion of the particle, it has limitation regarding the detection speed and sensitivity. Therefore, split-detection was performed by focusing the 532 nm laser onto the trapped sphere. The scattering light was divided into two parts by a D-shape mirror in *x*- and *y*-axis. In either direction, each half of the light was directed to a balance photodiode detector (QPD 450C, Thorlabs) and the variance of the two halves was compared to derive the position of the sphere. The split-detection scheme measures the position of sphere with higher sensitivity and speed, so it was used to acquire the sphere’s motion data. More details can be found in the Supplementary Information Section.

## 3 Theory

### 3.1 Mechanism of orbital-translational coupling

To explain the dynamics of the levitated particle in a transverse rotating optical trap, we conducted a theoretical analysis and numerical simulation of a homogeneous silica sphere with experimental parameters. Complete calculations of these quantities can be found in the Supplementary Information Section, the basic results are quoted and discussed below.

The motion of a levitated particle in a moving trap follows Langevin equation [38].

(1) $$\begin{array}{c} \;m\ddot{r}+\Gamma_{0}\dot{r}+\kappa \left(r-r_{\text{trap}}\right)-mg\hat{z}=f^{l}\left(t\right)\\ -m\omega^{2}R+i\omega \Gamma_{0}R+\kappa \left(R-R_{\text{trap}}\right)=F^{l}\left(t\right) \end{array}$$

**Figure 2:**
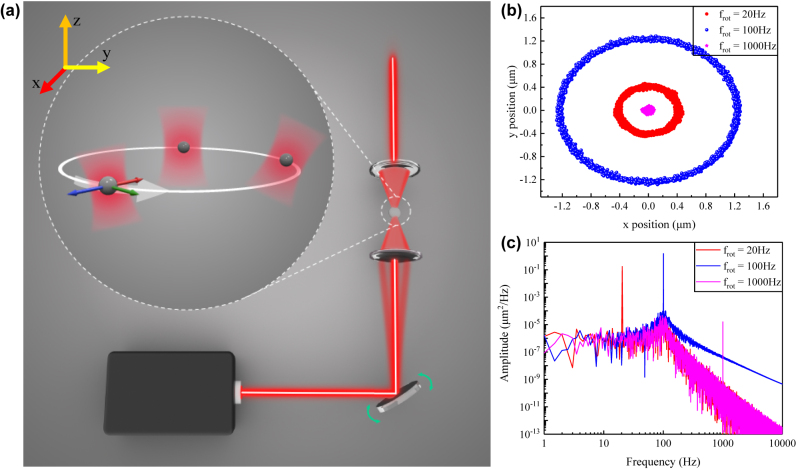
Illustration of an orbiting levitated particle in a rotational optical trap and typical simulated trajectories and power spectrum densities (PSDs) of an orbital microsphere. (a) Schematic of an orbiting levitated particle in a rotational optical trap caused by periodically tilting the incident optical beam. (b) Scatter plots of the orbital particle position distribution under various rotational frequencies. (c), PSD of the *x*-coordinates of levitated particle, corresponding to the simulation in (b).

The first expression denotes the equation of sphere motion in time domain, where *m* denotes the mass of the sphere, Γ_0_ denotes the damping rate, relating to the frictional force exerted on the microsphere, ***r*** denotes the position of the sphere, ***r***_**trap**_ denotes the position of the optical trap, and $f^{l}\left(t\right)$ denotes the stochastic Brownian force which has a zero-mean amplitude over a long period of time and represents thermal fluctuation. The second expression is the equivalent dynamics expression in the frequency domain, with ***R*** and ***R***_**trap**_ denoting the Fourier transform of ***r*** and ***r***_**trap**_, respectively. Although this model is idealized—that is, ignoring the inhomogeneous interior of the sphere, the fluctuation of laser noise, etc. —the results can still offer guidance to determine qualitative features of an optical trap system such as the mass of levitated particle. Specifically, Figure 2(b) and (c) show the simulated COM distribution and power spectral density (PSD) of levitated particle in a typical transversely rotational trap.

The introduction of an orbital degree of freedom is key to orbit levitated particle. The mechanism of an orbiting levitated particle in a rotational optical trap is shown in Figure 2(a). The essential feature here is the creation of a rotational trap using a periodically tilting incident beam. The axes shown in Figure 2(a) depict the orbiting process and axes coordinates used throughout this paper. The *x*- and *y*-axis denote two perpendicular directions on horizontal plane as well as the orbital plane. The *z*-axis denotes the axis along the direction of gravity as well as the direction of beam propagation. Detailed of experimental setup can be found in Methods and Supplementary Information Sections.

The transversely rotated trap can be denoted as: $r_{trap}=A\left(\hat{x}\cos\omega_{0}t+\hat{y}\sin\left(\omega_{0}t+\pi /4\right)\right)$ , with orbiting radius A and frequency *ω*_0_. The key feature here is the origin of the orbital degree of freedom of levitated particle originates from a transversely rotating optical trap. Such a scheme offers two practical benefits: First, a transversely rotating trap can be easily realized experimentally. In a converted optical trap system, a tilting incident beam naturally results in a transversely rotating optical trap. Second, the tilting angle of the beam can be flexibly controlled using a steering mirror with precise phase and amplitude control, providing excellent control over the orbiting degree of freedom.

Due to the thermal disturbance of the system, the dynamics of the levitated particle are mostly analyzed in the frequency domain. The second expression in Equation (1) is the equivalent expression in the frequency domain, with ***R*** and ***R***_**trap**_ denoting the Fourier transform of ***r*** and ***r***_**trap**_. At thermal equilibrium state, the PSD of an orbital microsphere along the *x*-axis can be defined as follows:

(2) $$X=\left\{\begin{array}{c} \frac{2k_{B}T\Gamma_{0}}{{\left(\kappa_{xx}-m\omega^{2}\right)}^{2}+\omega^{2}\Gamma_{0}^{2}}\omega \neq \omega_{0},\\ \frac{{\left(\sqrt{2k_{B}T\Gamma_{0}}+A\kappa_{xx}\right)}^{2}}{{\left(\kappa_{xx}-m\omega^{2}\right)}^{2}+\omega^{2}\Gamma_{0}^{2}}\omega =\omega_{0}\cdot \end{array}\right.$$

where *X* denotes the motion of the sphere in the frequency domain, *k*_*B*_ denotes the Boltzmann constant, *T* denotes temperature surrounding the sphere, *A,* ω0 denote the orbital radius and frequency of optical trap, *κ*_*xx*_ denotes the stiffness along *x* axis. The PSD provides a unique perspective of the sphere’s motion. For frequency domains except the orbital frequency, the levitated sphere randomly fluctuates as it behaves in a stable optical trap. At the orbital frequency, the levitated particle would orbit with the same frequency as the optical trap, but the radius changes along frequency due to the orbital-translational coupling within an optical trap. One prerequisite of this simulation is that the particle cannot escape the optical trapping volume—if the movement is larger than the optical trap linear range, this process would fail.

To obtain an intuitive understanding of the sphere’s motion, we can conduct a dynamics simulation of the orbital particles. During the simulation, successive multiplication of Equation (1) is conducted to obtain the orbital dynamics in a rotational optical trap. Atmosphere pressure is considered and the rotational trap is set as the parameter used in experiments. The COM distribution and PSD of *x* coordinates were shown with three frequencies in Figure 2(b) and (c), respectively. The results reveal strong coupling between orbital and translational degrees of freedom, as predicted in Equation (2). Specifically, at a lower frequency, the orbit radius is almost the same as the radius of the rotational trap. Additionally, the orbiting radius of levitated particle is amplified near the oscillation frequency and decreases at higher frequency.

Intuitively, there are at least two phenomena we could use from the orbital-translational coupling effect. First, since the orbit amplitude is dependent on the properties of the harmonic potential as well as the rotational frequency, we can take advantage of the frequency-response to qualify the properties of the harmonic trap. By fitting the coupling or amplification factor based on the response in the frequency domain, the properties of the optical trap including stiffness and viscosity can be derived. Additionally, as long as the rotational trap amplitude is larger than equivalent thermal force, the PSD of an orbiting microsphere will exhibit an ultra-narrow linewidth in the PSD curve, potential to be used as a micro-gyroscope with ultrahigh quality factor. If we exploit the orbital-translational amplification effect, the particle reaches its maximum orbiting diameter at the oscillation frequency. Consequently, an ultrastable levitated micro-gyroscope with maximum inertial momentum can be generated, with potential to be a sensitive angle detector. At a higher rotational frequency, the orbital-translational interaction can also be used to create a smaller orbit radius at diffraction limited scale.

## 4 Results

In this paper, we introduce an alternative orbiting method that allows for flexible speed and trajectory orbital control of a levitated particle. Previous rotational methods have used the offset or misalignment of two beams to realize the revolution or angular momentum to spin a sphere [27–29]. However, such an approach offers limited control over the rotational trajectory and frequency and requires sophisticated processes like nanofabrication and construction of structure light. This severely constrains the application range and a deeper understanding of the coupling effect in an orbital-translational system.

Alternatively, in a rotational optical trap, the orbit of a levitated particle is dependent on the properties of rotational trap such as the rotational frequency and amplitude. The momentum coupling between the trap rotation and its natural harmonic potential allows for flexible orbital control of the sphere. The key concept here is that the orbital trajectory of the sphere can be seen as phase-locked to the rotating trap, while its orbiting radius determined by the coupling efficiency and trap radius at given rotational frequency. As such, manipulation over rotating radius and frequency will be the key to improve the orbital degree of freedom of a levitated particle.

### 4.1 Flexible orbiting of levitated microsphere

Rotational manipulation of the optical trap has an easy realization in experiments. In a typical inverted-microscope optical trap, the trap location can be determined by the tilting angle of incident beam before the focus lens. By periodically changing the propagation angle of the incident beam using a field-programmable gate array (FPGA)-controlled steering mirror, a transversely rotating beam focus (thus an orbital microsphere) can be generated.

**Figure 3:**
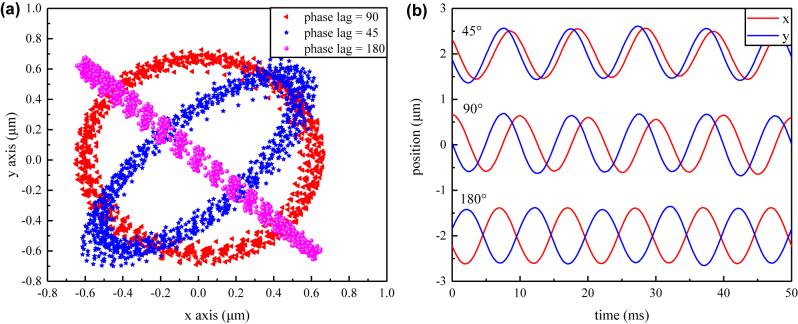
Experimental results showing flexible control of the orbital trajectories of various phase lags in the *x* and *y* signal. (a) COM position distribution in the *x*–*y* plane. (b) COM position along *x* and *y* direction in time domain.

In experiments, a microsphere of 25 μm diameter was levitated in an optical trap using a vertical optical trap. In order to create a large trapping volume, we used a weakly-focused laser beam with a nominal numerical aperture (NA) of 0.03, corresponding to a calculated beam waist of 11 μm. Such an approach provides excellent control over the amplitude as well as the frequency of the particle orbital mode. The orbiting trajectory of the sphere can be described by its COM position on the *x*–*y* plane, as shown in Figure 3.

To test the performance of our flexible orbital sphere scheme, we tracked the orbital motion of a levitated particle using various control signals along the *x* and *y* axes. An FPGA was used to provide two sinusoidal drives for independent control over the *x* and *y* axes, respectively. The steering mirror was monitored to oscillate at a frequency of 100 Hz with a tilting range of 4 μrad, corresponding to a rotational optical radius of 400 nm in our experimental setup. A varying phase lag of 45°, 90°, and 180° between the two axes allowed us to create circular, elliptical and linear trajectories of the sphere position, as shown in Figure 3(a). Figure 3(b) shows positions along the *x* and *y* axes, respectively, as a function of time.

### 4.2 Coupling with the harmonic potential

By rotating the optical trap, one can observe coupling over orbital-translational degrees of freedom of the levitated particles.

**Figure 4:**
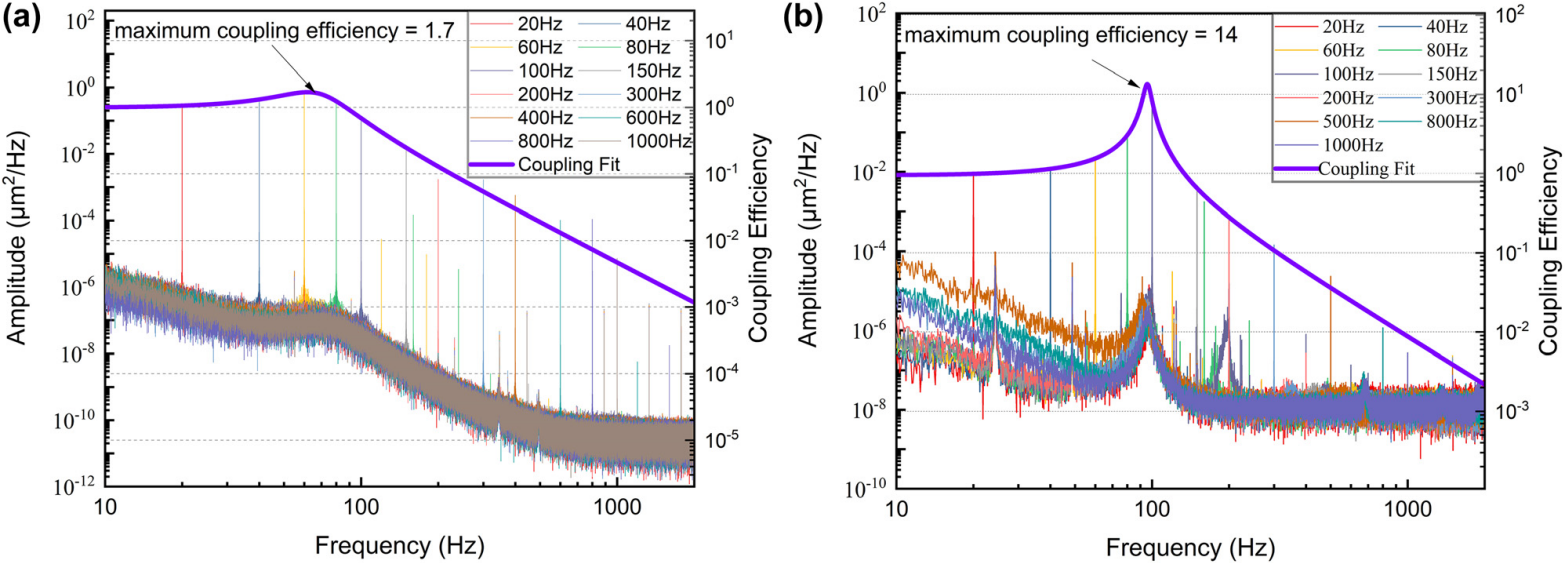
Orbital-translational coupling effects as a function of rotational frequency under two pressures. The PSD signals of the *x* coordinates using optical trap rotating at various rotational frequencies under (a), atmospheric pressure and (b), 1 mbar pressure.

To study frequency-dependent coupling effects, a series of experiments using various orbiting frequencies were conducted. The orbital motion of a levitated particle was tracked and its PSD signal along the *x* axis was shown in Figure 4. By trapping and orbiting a microsphere at two pressures, we could observe frequency-dependent interaction over various degrees of freedom, determined by the harmonic potential and environmental viscosity. Such an optically trapped and simultaneously rotated particle offers an original perspective on rotational opto-mechanics and orbital-translational coupling.

Experimentally, tilting signal along the *y* axis had a 90° phase lag after the *x* axis, making sure that the sphere moved in a circle. A series of experiments were conducted over an orbital frequency range of 20–1000 Hz. We measured the position of the sphere over the *x* axis and calculated its PSD, as shown in Figure 4. Figure 4(a) shows the frequency-dependent orbital-translational coupling at atmospheric pressure, at a laser power of 500 mW and frequency range of 20–1000 Hz. The results show a remarkable dependency between the orbital and harmonic potential of the trap. Intuitively, the orbital particle reaches its maximum coupling efficiency at the oscillation frequency. An obvious drop in the orbiting amplitude was indeed observed in our experiments as the rotational frequencies increase. As predicted in Equation (2), the sphere could attain a larger orbiting radius at lower pressures, so we conducted comparative experiments in a lower viscosity environment at 1 mbar pressure, as shown in Figure 4(b). Compared with the orbital results at atmospheric pressure, the microsphere orbiting radius is greatly magnified near the oscillation frequency, implying a stronger coupling effect at lower pressures.

Based on orbital dynamic analysis, the orbital response of the levitated particle could be exploited to obtain the optical trap properties. In Figure 4, we fit the amplitude of the PSD response peak at the rotational frequency using Equation (2). Thus, we can obtain the coupling efficiency factor (shown in right *y* axis) and calibrate the mass of the levitated particle. In our fitting, we use a calculated viscosity of 18 μPa s at atmosphere and 1.98 μPa s at 1 mbar [39]. According to the measured orbital response, the mass of the levitated particles in Figure 4(a) and (b) were 27.3 ± 2.22 ng and 39.6 ± 11.6 ng, respectively.

### 4.3 Orbital rotor with extreme values

Previous results revealed that the orbital trajectory of a levitated particle was determined by the motion of the rotating optical trap as well as orbital-translational coupling. Notably, the sphere could achieve its largest orbiting amplitude as the orbital frequency *f*_rot_ coincides with the oscillation frequency *f*_osc_. This implies the levitated orbital rotor realized its maximum energy flow when orbiting at the oscillation frequency. Such a process could offer unprecedented opportunities to generate a levitated micro-gyroscope with a large orbital radius, indicating large angular inertial.

Based on the rotating properties revealed in Equation (2), the orbital particle could achieve a high quality factor as long as the rotational trap has a larger orbital radius than the effective thermal fluctuation, determined only by the external drive quality factor. This remarkable effect is due, in part, to the orbital motion of the sphere is adhering to the trap position. Consequently, it can achieve the same high quality factor as the parametric drive, which can be synchronized to a high standard time drive.

We then investigated the possibility of creating an ultra-stable micro-gyroscope with a large inertial angular momentum by exploiting orbital-translational coupling. As discussed previously, the particle achieved its maximum orbital momentum transfer at the oscillation frequency, so the rotational frequency was first set to be the same as the oscillation resonance of approximately 100 Hz.

**Figure 5:**
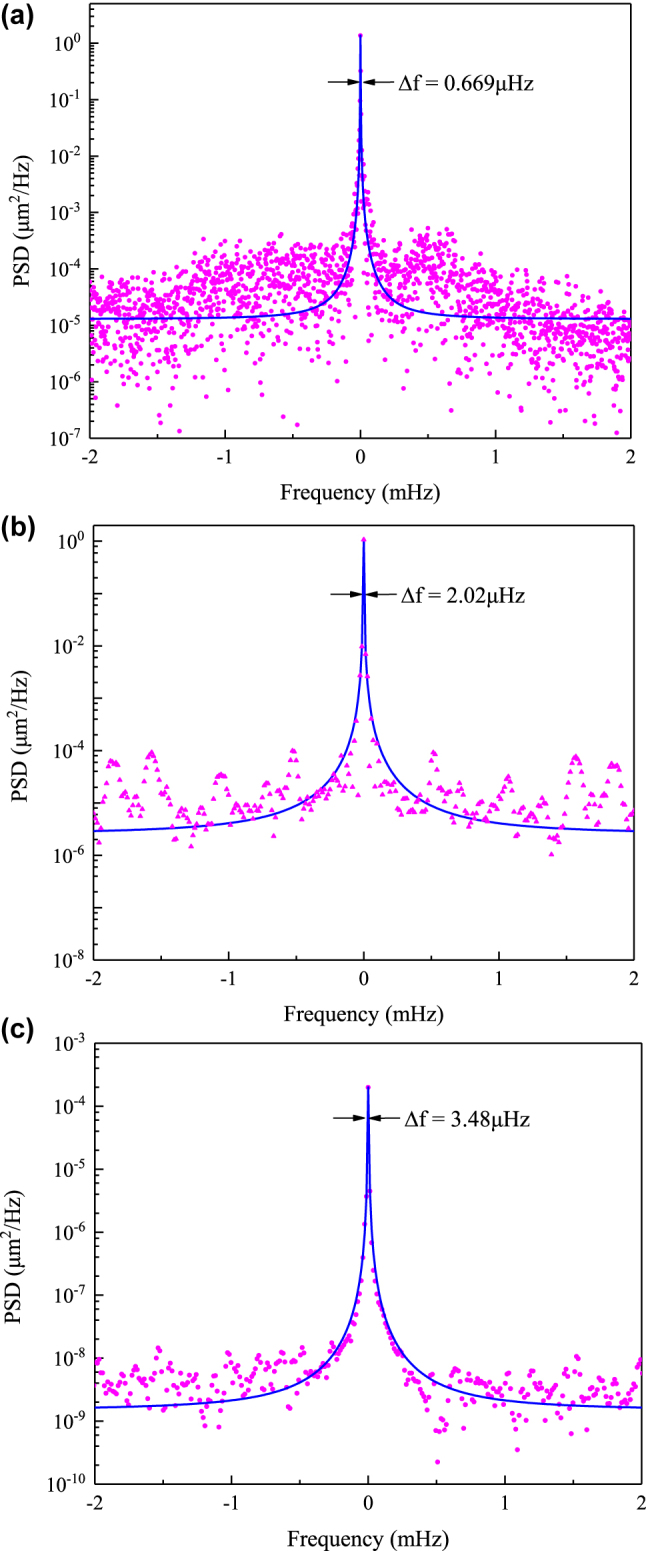
The PSD signals under various orbiting conditions and experimentally measured PSD signals fitted with Lorentzian function of the orbital particle at various modes. (a) 100 Hz orbiting PSD signals at low pressure (1 mbar) showing a linewidth of 0.669 μHz, yielding a quality factor of *Q* = 1.47 × 10^8^. (b) 100 Hz PSD orbiting signal at atmosphere pressure with 2.02 μHz and *Q* = 4.95 × 10^7^. (c) 500 Hz orbiting signal at low pressure (1 mbar) with 3.48 μHz linewidth and *Q* = 1.43 × 10^8^.

Figure 5(a) shows the PSD of the orbital particle along the *x* axis. The results were obtained under 1 mbar pressure; the steering mirror orbits provide a 100 Hz rotational signal for more than 50 h. The x coordinates of the orbital particle were continuously measured and its PSD calculated. As can be seen, the orbital mode of the sphere has an ultranarrow linewidth of Δ*f* = 0.669 μHz, corresponding to a quality factor of *Q* = 1.47 × 10^8^. To test the applicable range of the gyroscope’s long-term stability, we tested the stable orbiting mode under atmosphere pressure at 100 Hz rotational frequency. Figure 5(b) shows the orbital mode of the sphere has a linewidth of 2.02 μHz at 100 Hz rotational frequency, corresponding to *Q* = 4.95 × 10^7^. Such a parameter can be improved by increasing the measurement time (currently 17 h). Our scheme could also be extended to generate an orbital rotor to other rotational frequency. Figure 5(c) represents a levitated particle orbiting at 500 Hz under low pressure (1 mbar) with a 3.48 μHz linewidth and *Q* = 1.43 × 10^8^. As the particle rotates, the orbital-translational coupling allows us to create an orbital rotor of approximately 10 nm orbit radius. These are, to the best of our knowledge, the smallest orbiting radius and highest quality factor micro-gyroscope reported to date for an optically levitated particle.

## 5 Conclusions

In this study, we demonstrated the novel control of a ring-shaped levitated rotor with the highest quality factor (Q) and smallest orbiting radius by means of orbital-translational coupling. The levitated particle achieved its orbital degree of freedom from the rotational optical trap, permitting flexible control of the orbiting process to be accessed. A simple model was developed to describe this coupling process and to determine the properties of the optical trap. However, only the ideal situation was considered, and ongoing work is needed to consider the more practical impact of the inhomogeneous and nonlinearity interior of the optical trap and the coupling within the axes.

This system could provide new possibilities in generating levitating orbital rotors with extreme parameters. For example, an orbital micro-gyroscope system with larger angular inertial momentum could be generated, providing the optical trap could be rotated within a larger circle. An orbital rotor of sub-nanometer scale could also be achieved through orbital-translational coupling at a higher frequency, which could reveal the light–matter interaction at a sub-diffraction scale [30]. Another exciting aspect concerns the generation of an orbital rotor with a higher quality factor. Because the tilting speed of the steering mirror and the large inertial momentum of the microsphere, orbital frequencies in the MHz range have not yet been explored. However, the above hurdles could be overcome using an acoustic-optical modulator (with a MHz tilting frequency) and the rotation of a nanosphere (which has an oscillation frequency of hundreds of kHz) to achieve a measurable orbital-translational response in the MHz range [40]. An ultrahigh rotational quality factor of up to 10^12^ could be achievable.

Adding an orbital degree of freedom to optomechanics could be highly advantageous in studying the coupling effects over various degrees of freedoms at mesoscopic regime. Combined with existing methods for controlling the translational and spin degree of freedom, adding orbital degree provides opportunities to study the coupling within orbital, spin, and translational degrees of freedoms and offers new research possibilities in stochastic thermodynamics. Additionally, with recent advances showing an optically levitated particle to have been cooled down to a quantum ground state, the observation of a macroscopic quantum superpositions could become achievable, providing the quantum orbital state or spin state can be generated.

Finally, the controllable orbital state offers new possibilities in the life sciences [41, 42]. Since our method could be applied to all trappable objects, precise control over bacteria and cells could be realized. Consequently, biological activities and the response of orbital motion could be studied.

## Supplementary Material

Supplementary Material Details
