# Supplementary material for: Flexible control of an ultrastable levitated orbital micro-gyroscope through orbital-translational coupling
Source: Nanophotonics. 2023 Feb 28;12(7):1245–53. doi: 10.1515/nanoph-2022-0625 (PMC11636352; doi:10.1515/nanoph-2022-0625)
Supplement: Supplementary file 1 — Supplementary Material Details [file j_nanoph-2022-0625_suppl.docx]

**Supplementary Information**

**Flexible Control of an Ultrastable Levitated Orbital Microgyroscope through Orbital-Translational Coupling**

**Wenqiang Li^1,*^, Xia Wang^1^, Jiaming Liu^1^, Shuai Li^2^, Nan Li^1,*^, Huizhu Hu^2,3,*^**

*^1^College of Optical Science and Engineering, Zhejiang University, Hangzhou 310027, China*

*^2^Quantum Sensing Center, Zhejiang Lab, Hangzhou 310000, China*

*^3^State Key Laboratory of Modern Optical Instrumentation, Zhejiang University, Hangzhou 310027, China*

*Corresponding author: [liwenqiang@zju.edu.cn](mailto:liwenqiang@zju.edu.cn) [nanli@zju.edu.cn](mailto:nanli@zju.edu.cn) [huizhuhu@zju.edu.cn](mailto:Huhui%20Zhu@zju.edu.cn)

1. Theoretical Modeling and Simulation

1.1. Optical force calculation based on ray optics methods

Key to simulate the experimental dynamics of a levitated particle is obtaining an accurate model of how the optical forces are exerted on the sphere. Considering the large size of the particle used in our experiments, the ray optics method was suitable to calculate the optical force field in a Gaussian beam optical trap, whose accuracy has been proven in our previous study [1]. To analyze the coupling effect of the orbital dynamics of the particle and harmonic potential, a weakly focused Gaussian beam was generated to ensure a large trapping volume and large motion range. Below is a brief description of the investigative model and method used. The trapping beam was weakly focused using a lens of low numerical aperture. Moreover, at the focus point, the Gaussian beam could be regarded as a paraxial beam.

The mathematical expression for the electric field amplitude is a solution to the [paraxial Helmholtz equation](https://en.wikipedia.org/wiki/Helmholtz_equation#Paraxial_approximation) [2]. Assuming polarization in the *x* direction and propagation in the *z*-direction, the electric field in [phasor](https://en.wikipedia.org/wiki/Phasor) (complex) notation can be expressed as follows:

where *r* denotes the radial distance from the center axis of the beam, z denotes the axial distance from the beam focus (or beam waist), *i* denotes the imaginary unit, *K* = 2π/λ denotes the wave number (in rad/m) for a wavelength *λ*, R(z) denotes the radius of curvature of the wavefront at *z*, and E_0_ = E(0,0) denotes the electric field amplitude (and phase) at the center of the beam focus.

**Figure S1**. Geometric illustration of the ray optics calculation method. A dielectric sphere located at O is illuminated by a Gaussian beam originating from point O_1_. The beam waist is parallel to the *x-y* plane. The beam axis O_1_A is deviated from the *z-*axis by a distance *d* along the axial direction.

By decomposing Gaussian beams into separate rays, we can obtain the direction and power of each ray and the optical force being exerted on the sphere, as shown in Fig. S1. Since the position and radius of the sphere can be calculated, it is possible to calculate the optical force originating from a single ray. Then, by computing the integral of all rays hitting the sphere surface, we can obtain the overall optical force. The axial and transverse optical force in a typical Gaussian filed can be expressed as follows [3]:

where and are defined as trapping efficiency relating optical force and trapping laser power P. $R$ is radius of levitated particle. $\theta$ and are polar and azimuthal angles of the hitting point P on the microsphere, respectively. $\omega$, *R_c_* and *R_z_* are beam size, the radius of curvature and axial position at hitting point P (In Fig. S1). *R_z_* and *d* reoresebt the axial and radial position of hitting point P. The angle $\gamma$ is angle between the optical ray direction and the position of the sphere. $q_{s}$ and $q_{s}$ are the fractions of the momentum transfer factor as an incident ray hit microsphere in the direction parallel to the incident ray and perpendicular to the incident ray, respectively. They can be expressed as:

$$q_{s}=1+R\cos2\alpha_{i}-T^{2}\frac{\cos(2\alpha_{i}-2\alpha_{r})+\mathrm{Rcos}2\alpha_{i}}{1+R^{2}+2R\cos2\alpha_{r}}$$

$$q_{g}=-R\sin2\alpha_{i}+T^{2}\frac{\sin(2\alpha_{i}-2\alpha_{r})+\mathrm{Rsin}2\alpha_{i}}{1+R^{2}+2R\cos2\alpha_{r}}$$

Where R and T are the reflectance and transmittance at the surface of the microsphere. $\alpha_{i}$ and $\alpha_{r}$ represent the incident angle and refractive angle between the incident ray and microsphere.

Using the typical parameters used in experiments, a microsphere of radius 25μm was illuminated by a Gaussian beam with 20μm waist was considered. Its axial and radial optical forces was calculated as shown in Fig. S2. To have a comparison of the experimental results we conduct simulation using the nominal parameter in experiments.

**a**

**b**

**Figure S2**. Typical simulation results of optical force along the (**a**) transverse and (**b**) axial directions.

1.2. Dynamics simulation

Direct simulation of the particle motion in a movable optical trap can be based on the Langevin equation, which can be expressed as follows:

$$m\text{r}+\Gamma_{0}\text{r}+\kappa\left(\text{r-r}_{\text{trap}}\right)-mg\hat{z}=f^{l}\left(t\right)$$

where ***r*** denotes a three-dimensional parameter indicating the center of mass (COM) of the levitated particle, $\text{r}_{\text{trap}}$ denotes the location of the optical trap, *M* denotes the mass of the sphere, $\kappa$ denotes the optical stiffness, $\Gamma_{0}=6\pi\mu r$ denotes the damping rate of the COM motion of a microsphere particle radius *r* in a solution of dynamic viscosity $\mu$, and $f^{l}\left(t\right)$ denotes the Langevin force originating from air stochastic collisions, which has a mean value of zero.

$$\begin{matrix} \left\langle f^{L}\left(t\right)\right\rangle=0 \\ \left\langle f^{L}\left(t\right)\otimes f^{L}\left(t\right)\right\rangle=2k_{B}T\Gamma_{0} \end{matrix}$$

The trajectory of trapped particle motion was measured using the integration scheme described in Ref. [4]. Although this numerical simulation only considered translational motion in a stationary optical trap, it could also be extended to rotational traps by adding a rotational parameter $\text{r}_{\text{trap}}$ in equation **.** In the dynamic analysis process, the dynamics of the levitated particle are expressed by a series of states involving the particle position and velocities in successive timesteps. During each timestep, the current sphere dynamic state was determined by the previous state and a random thermal force, resulting in a unique particle motion trajectory for each simulation. Consequently, the dynamics of the sphere could be deduced through successive integration of the particle motion state.

Table S1 describes the numerical parameters used in the simulation, corresponding to the nominal parameters used in the experiments. In our simulation, the radius of the microsphere was 25 μm, regarded as a homogeneous pure silica sphere of refractive index 1.45 and density 2560 kg/m^3^. An optical power of 500 mW was used, the sphere having an oscillation frequency of ~100 Hz along the *x-* or *y-* axes under such conditions. As the simulation ran, a Brownian trajectory could be observed. From this, we could evaluate the power spectra to obtain additional properties of the harmonic potential.

**Table S1**. default parameters used in experiments and simulation.

| Quantity | value |
| --- | --- |
| Laser power | 500mW |
| Beam wavelength | 1064nm |
| Beam waist | 11μm |
| Sphere radius | 25μm |
| Refractive index | 1.45 |
| Time step | 1us |

A rotational optical trap was used to introduce an orbital degree of freedom to the levitated particle. The optical trap rotated in a circular orbit, the position of which could be approximately expressed as$r_{trap}=A\left(\hat{x}\cos\omega t+\hat{y}\sin\left(\omega t+\pi/4\right)\right)$, where $A=400nm$ was set as the rotational radius of the optical trap and $\omega=2\pi f_{\text{rot}}$ was the angular rotational frequency of the rotational trap. In the simulation, two conditions—namely, atmospheric and low-vacuum pressure—were considered. Figure S3 shows the motion trajectories of the COM in a rotational optical trap of various rotational frequencies. The figure shows the orbital position distribution, power spectrum density (PSD) of the *x-*coordinates and the coupling efficiency at three rotational frequencies. One thing to mention is the rotational radius is 100 nm at 1 mbar (100 Hz) because interaction between the harmonic potential and rotational movement of the optical trap pushes the sphere out of the trapping volume. Apart from the condition at 1 mbar (100 Hz), the rotational optical radius was set to 400 nm for all simulations.

The simulations afforded us an intuitive understanding of the orbital motions of the particle. The scatter coordinates (*x, y*) radius difference implied various interaction efficiencies between the rotational optical trap and harmonic potential. The result implied that the coupling efficiency was clearly influenced by the rotational frequency as well as the environmental viscosity.

1.3 Theoretical performance of orbital-translational coupling

In optomechanical dynamic analysis, the power spectrum is a common method used to analyze the dynamic properties of a levitated particle. Here, we outline the orbital dynamics in the frequency domain and analyze the orbital-translational coupling efficiency. The theoretical analysis can be started by transferring the Langevin equation into the frequency domain, as follows:

$$m\text{r}+\Gamma_{0}\text{r}+\kappa\left(\text{r-r}_{\text{trap}}\right)-mg\hat{z}=f^{l}\left(t\right)\Leftrightarrow m\text{R}+\Gamma_{0}\text{R}+\kappa\left(\text{R-R}_{\text{trap}}\right)=\text{F}^{\text{l}}\left(\text{t}\right)$$

where ***P***，***R***, and $F^{l}\left(t\right)$ denote the Fourier transform of the corresponding part in the left-hand expression of equation (S8). Since we create an orbital degree of freedom on the *x-y* plane, we focus on the Langevin equation in the *x-y* plane to simplify the analytical process. The parameters can be expressed in matrix form as follows:

$$\kappa=\left[\begin{matrix} \kappa_{xx}\text{ }\kappa_{xy} \\ \kappa{}_{yx}\text{ }\kappa_{yy} \end{matrix}\right],r=\left(\begin{matrix} x \\ y \end{matrix}\right),r_{opt}=\left(\begin{matrix} a\sin w_{0}t \\ a\cos w_{0}t \end{matrix}\right)$$

where $\kappa$ denotes a stiffness matrix consisting of the trap stiffness $\kappa_{xx}$ and $\kappa_{yy}$ along the *x-* and *y-* axes, while $\kappa_{xy}$ and $\kappa_{yx}$ denote the coupling factor within the stiffness coefficients. It should be noted that when considering an optical trap created using a Gaussian beam, the optical force along any transverse direction is axisymmetric and proportional to the displacement of the microsphere. Consequently, the coupling factor can be ignored as $\kappa_{xy}=\kappa_{yx}=0$ in a harmonic optical trap. The above Langevin equation can be simplified in one dimension, the power spectrum density along the *x-*axis being expressed as follows:

$$X=\left\{\begin{matrix} \frac{2k_{B}T\Gamma_{0}}{\left(k_{xx}-m\omega^{2}\right)^{2}+\omega^{2}\Gamma_{0}^{2}} \\ \frac{\left(\sqrt{2k_{B}T\Gamma_{0}}+Ak_{xx}\right)^{2}}{\left(k_{xx}-m\omega^{2}\right)^{2}+\omega^{2}\Gamma_{0}^{2}} \end{matrix}\right.$$

According to equation , the frequency domain of the orbital dynamics comprises two parts. For most frequency domains except the orbiting frequency, the power spectrum of the COM motion is the same as in a normal stationary optical trap. However, at the rotational frequency of the optical trap, if the drive is comparatively larger than the thermal fluctuation force, the motion at the rotational frequency is dominated by the rotational drive and exhibits a sharp peak at the rotational frequency. Additionally, the particle movement orbit is determined by the frequency and viscosity simultaneously. As such, interaction between the harmonic potential and the rotation of the optical trap can be observed and is determined by the drive frequency and viscosity. Oscillations in the x- and y-direction couple with the rotation of the optical trap. The amplitude and phase response can then be calculated.

**c**

**b**

**a**

**1bar**

**1mbar**

**Figure S3.** Simulation data showing the orbital position of the levitated particle at various pressures. (**a**) The PSD of the *x*-coordinate at three rotational optical trap frequencies. (**b**) A scatter plot of the position distribution of the levitated particle position. (**c**) The frequency dependency on coupling efficiency, which can be defined as the rotational radius amplification factor ($R_{sphere}/R_{trap}$).

In Fig. S3, the top figures show the coupling effect under atmospheric pressure. The lower plots show the results under a pressure of 1 mbar. Both conditions represent the simulated results of the PSD (Fig. S3(a)), COM distribution (Fig. S3(b)) and coupling efficiency (Fig. S3(c)). The coupling efficiency can be defined as the orbiting radius amplification factor, also denoted by $R_{sphere}/R_{trap}$, to qualify the energy transfer efficiency between the orbital and translational degree of freedom. Additionally, the coupling phenomena under the two pressures were simulated, a larger coupling efficiency being observed under the lower pressure.

2. Experimental setup and detection scheme

**Figure S4.** The optical setup used for the trapping and orbiting of a levitated particle in a vacuum chamber.

A schematic of the experimental apparatus is shown in Fig. S4. We built a vertical optical trap to levitate a silica particle. A linearly polarized laser beam of wavelength 1064 nm was used in the experiments at a maximum laser output power of 5 W (YFL-SF-1064-10-CW, Precilasers Technology). The output beam was later tilted using a steering mirror (PSM2, Newport) in the horizontal *x-y* plane, at a maximum tilting frequency of 5000 Hz and a range of 2 mrad. The tilted beam was focused using an aspherical lens with a focal point of 100 mm, corresponding to a nominal NA of 0.03 with an input beam size of 3 mm. In the microscope system, the tilting incident beam is transferred to a rotational beam focus, realizing a rotation optical trap, the optical field having a Gaussian mode with a 11 µm beam waist.

Such a weakly-focused optical trap has two primary benefits. First, the longer focal distance promises a larger travel distance for an optical trap with the same tilting angle—that is, the moving area of the microsphere. Second, the weakly-focused Gaussian beam can create an optical trap with a larger trapping volume, which allows the microsphere to orbit in a larger circle than in a rotational optical trap, allowing us to study orbital-translational coupling when the microsphere orbit is larger than that in a rotational optical trap.

Since the trapping laser moves in our experiments, it is unsuitable for use as the detection laser. Consequently, to measure the orbital trajectory of the sphere, an additional 532 nm laser was introduced into the optical trap and used for detection. It was colinear with the trapping laser, by means of using a dichromic mirror. A pair of identical collimation lenses were set in the detection path to control the divergence angle of the detection beam, and ultimately, the beam illumination area at the trapping plane. The controllable illumination size allowed us to detect over a large area since the orbital trajectories of the microsphere change from nano to sub-millimeter scales. Specifically, two detection methods were used in our experiments for various detection and precision measurements by splitting the detection beam into two parts. An aspherical lens (condenser) was used to collect the scattering light and the trapping laser beam was attenuated using a short-pass filter. The detection beam could be split into two parts to be used to conduct either the split-detection method used in Ref. [5] or to serve as an illumination light in the microscope image method. In the microscope image scheme, the lens pair was set to generate an appropriate divergence angle to create a large illumination area at the trapping plane. The transverse images were captured using a CCD camera at 25 frames/s. The image detection scheme had a large detection area, offering intuitive detection of the sphere motion. A more precise detection scheme was the split-detection method, where the collimation lens pair was set to generate the collimation beam, which could be focused to hit the levitated particle at the trapping plane. The scattering light could then be divided into two parts using a half-reflection mirror (D-shape mirror), as shown in Fig. S4, each half being directed to the balance photodiode detector (QPD 450C, Thorlabs), the variance of the two parts being compared to derive the position of the sphere. The split-detection scheme offers precision and a rapid detection speed. It is also used to track the motion of the sphere over the long term.

To create an orbital trajectory of the levitated particle, two sine wave functions were generated using an FPGA to control the *x*- and *y*- tilting angle of the steering mirror. We could change the phase difference between these two signals to manipulate the ovality of the rotational trap, and ultimately the orbital trajectory of the sphere. The same FPGA could also be used to collect the signal from the detection system.

3. Experimental Demonstration of Orbiting Levitated Particle

3.1. Flexible control of orbital levitated particle trajectory

Based on the dynamic simulation conducted in the previous section, the particle could be seen to be locked to the orbital movement of the optical trap. We could realize levitated particle orbits of arbitrary shape and speed by adjusting the tilting angle of the incident light. Here, we describe how we could control the trajectory of the levitated particle. By changing the phase of the steering mirror, circular, elliptical, and linear motion of the optical trap could be realized, thus the position of the levitated particle. The controlling process was performed using an FPGA-based controller. Two independent signals were output to the *x-* or *y-* axes to control the beam propagation direction along the two axes.

As discussed previously, the position of the sphere could be monitored using two methods, the more direct method being to record its motion using the microscope system. The time-lapse images of the orbital particle are shown in Fig. S5. The upper image shows a slow orbiting of 0.2 Hz about a radius of ~150 µm, while the lower image shows the results at a rotational speed of 100 Hz, the white lines and arrows indicating the trajectory and direction of the levitated particle, respectively. The length of the scale bars in the two images is 100 and 20 μm, respectively.


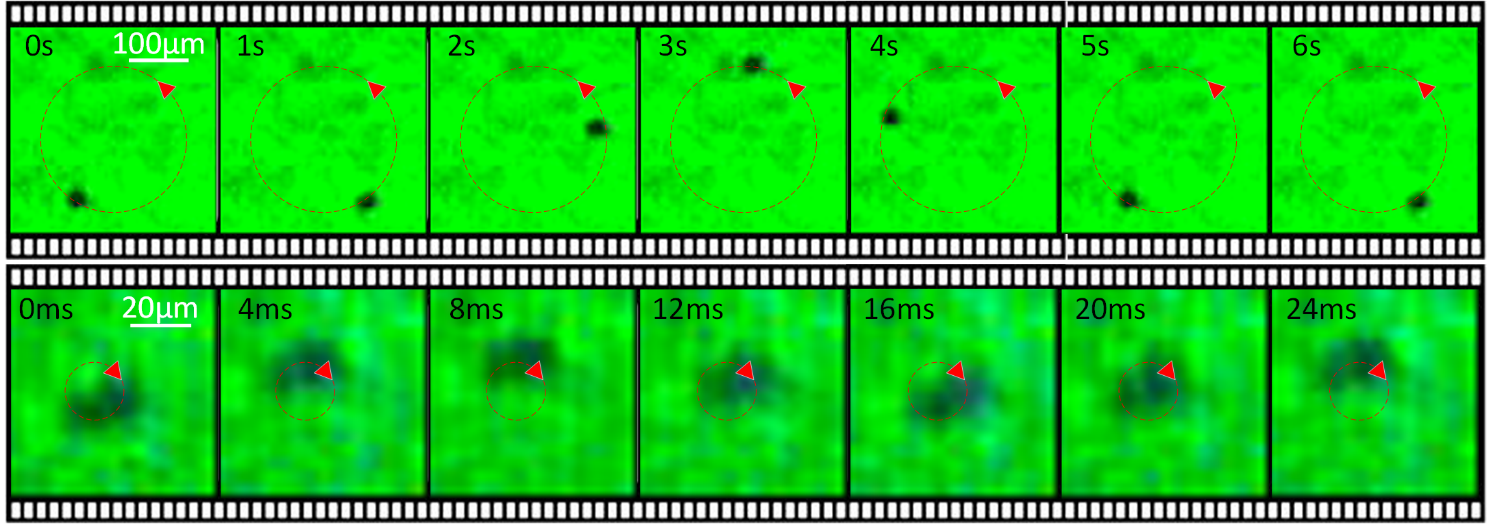


Figure S5. Successive frames of a movie recording, which show the rotation of a particle in a rotational optical trap at 0.2 and 100 Hz rotational frequency, respectively. The images were captured using a CCD camera at 25 frame/s, the red dashed lines and angle indicating the direction of motion.

Due to the NA limitation in the microscope system, small movements of particles of sub-micron scale cannot be detected. A more precise detection of the particle position is required to obtain its motion at sub-diffraction scale. Split-detection methods use scattering light differences to obtain the positional information of the particle. As shown in Fig. S4, the forward propagation beam is split using a D-shape mirror, disintegrating into two parts. Positional information of the sphere is contained in the variance between the two parts.

Both beams were introduced into a balanced 70 MHz photodetector (PDB450C, Thorlabs) and the output RF signal was digitized using an oscilloscope running in high-resolution mode at a 10 KS/s sampling rate.

Arbitrary trail control of the levitated particle was realized, as shown in Fig. S6. The left-hand image shows the positional distribution of the levitated particle in a rotational trap. The blue, red, and magenta scatters are trajectories with x-y phase lags of 45°, 90°, and 180°, respectively. Under these conditions, the movement range of the levitated particle was set to be 600 nm. The left image represents the x- and y- motion of the orbital particles, separately, making the motion position of the circular, elliptical, and linear lines accessible.

**b**

**a**

**Figure S6.** Experimental results showing the flexible control of orbital trajectories using various phases in the *x-y* tilting signal. (**a**) COM position distribution in the *x-y* plane, and (**b**) their *x-* and *y-* positions using different phase lags along the *x-* and *y*- axes.

3.2. Coupling of orbital and translational movement

According to the theoretical model discussed in Section 1, the orbital degree of freedom of the levitated particle can be determined by two factors. The first is the orbital size of the optical trap, which can be seen as the angular momentum input in the orbital-translational system. Its rotational speed and radius impart an orbital momentum to the particle. Additionally, the system is in an optical trap generated using a Gaussian beam. The harmonic potential in all dimensions would be naturally coupled with the orbital degree of freedom. Consequently, the coupling between the orbital degree of freedom and the harmonic potential could be observed by recording the sphere’s position.

The coupling effect predicted in Section 1 is well demonstrated in the experiments. One key feature is that at a lower frequency, the sphere orbital is the same as the rotational optical trap, the coupling efficiency increasing as the frequency increases. It achieves its maximum orbital size at the resonant frequency of the harmonic trap. As the rotational frequency increases, coupling with the harmonic potential restrains the orbital momentum transfer to the sphere, resulting in a smaller orbiting radius.

**b**

**a**


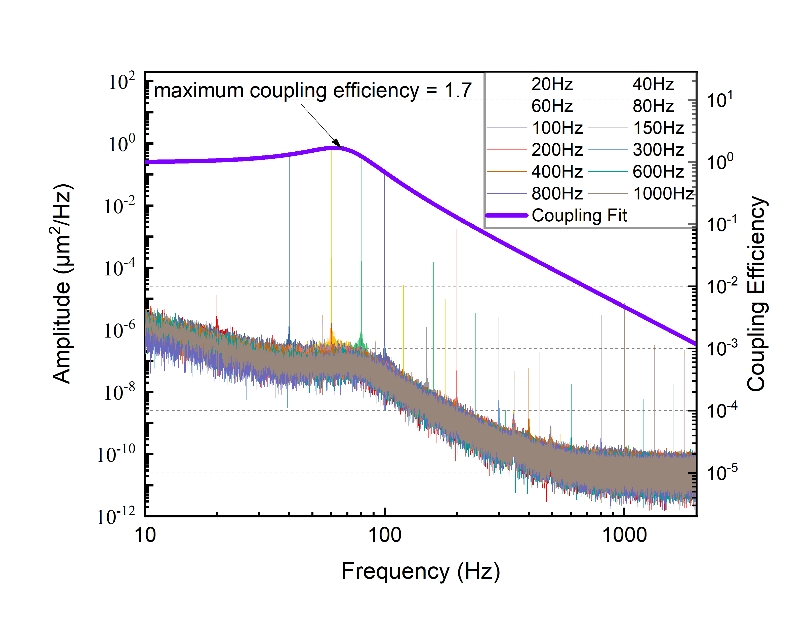

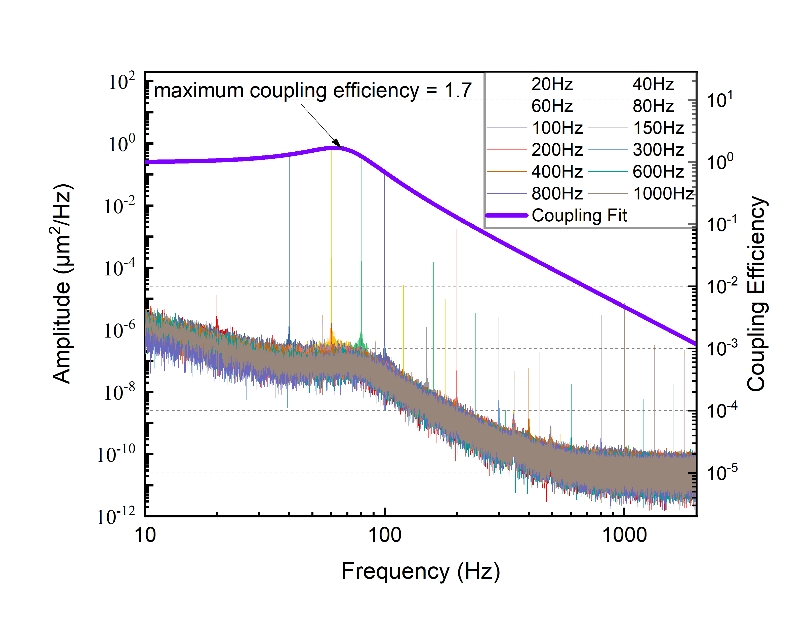


**Figure S7.** Experimental results for the orbital-translation coupling efficiency at various rotational frequencies under (**a**) atmospheric pressure, and (**b**) low pressure of 1 mbar.

The results of the coupled effects in our experiments are shown in Fig. S7 for two particles trapped at two different pressures. Each data is collected at a 5 kHz data acquisition speed and acquired for ~10 mins. In each figure, the same particle was trapped, and its orbital movements over various frequencies measured. The amplitude response over a frequency range of 20–1000 Hz was collected and analyzed by fitting the amplitude response to the theories expressed in equation . The coupling efficiency was also calculated in right y axis. The calculated mass of the levitated particle used in the two figures were $27.3\pm2.22ng$ and $39.6\pm11.6ng$, respectively.

3.3. Ultra-stable microscope with extreme values

Qualitatively, if the rotational optical trap radius is larger than the motion fluctuation induced by thermal forces, the power spectrum could exhibit a sharp peak at the orbital frequency, implying an ultrastable orbital rotor with a high quality factor. The ability to control the rotational optical trap realizes a flexible rotor with extreme values, such as an ultrastable orbital microscope or orbit at a sub-diffraction scale.

Here, we consider a common case in which the particle is driven by a circular rotational trap, the optical trap rotating in a circle to generate an orbital microsphere. The most obvious consequence of such system is that it could generate an ultrastable orbital microsphere as the quality factor of the orbital rotor is independent of the viscosity, merely being determined by the rotational stability of the optical trap, which is related to the quality of the controlling signal. Figure S8 shows the experimentally measured orbital PSD data of the *x-*coordinates, fitted using a Lorentzian curve (blue line) under three conditions.

Figure S8(a) shows the results acquired under low vacuum conditions (1 mbar), exhibiting a line width of 0.669 μHz at 100 Hz and corresponding quality factor of $1.47\times10^{8}$. These stable orbiting properties are also observed under atmospheric pressure conditions. Figure S8(b) shows the orbiting PSD data results under atmospheric pressure, while Fig. S8(b) shows that an orbiting frequency of 100 Hz yields a linewidth of 2.02 μHz, corresponding to a quality factor of $4.95\times10^{7}$. The amplitudes of the orbit radius in Fig. S8(a) and S8(b) are close because the optical trap orbiting radii are different in these two figures. At lower pressure, the coupling efficiency at oscillation frequency would be ~10 times larger than that at atmospheric pressure. If the optical trap orbits with the same radius at lower pressure, the orbital radius of the particle would be too large. This may lead to the escaping of the particle or nonlinearity effects in optical trap, so we decreased the optical trap orbiting radius at 1 mbar to avoid the drawbacks. Figure S8(c) shows the PSD at a 500 Hz rotation frequency under low vacuum conditions (1 mbar), yielding a linewidth of 3.48 μHz and quality factor of $Q=1.43\times10^{8}$. The results verify the accuracy of the simulation predictions discussed in Section 2. The PSD data acquired over 50, 17, and 19 h are shown in Fig. 8S(a)-(c), respectively

**b**

**a**

**c**

**Figure S8.** Experimental results showing the PSD of the levitated particle in a long-term stability testing mode. (**a**) 100 Hz orbiting signal under low pressure conditions (1 mbar) yielding a linewidth of 0.669 μHz, and a quality factor of $1.47\times10^{8}$.(**b**) 100 Hz orbiting signal under atmospheric pressure yielding a linewidth of 2.02 μHz, and a quality factor $Q=4.95\times10^{7}$. (**c**) 500 Hz orbiting signal under low pressure conditions (1 mbar) yielding linewidth of 3.48 μHz, and a quality factor $Q=1.43\times10^{8}$.

4. Additional supplementary materials

**Supplementary Movie 1** shows a silica sphere (25 μm in diameter) orbiting along the x-y plane with a radius of ~150 μm and frequency of 0.2 Hz. It is also the original video of Fig. S5(a) in this supplementary material.

**Supplementary Movie 1** shows a silica sphere (25 μm in diameter) orbiting along the x-y plane with a radius of ~10 μm and frequency of 100 Hz. It is also the original video of Fig. S5(b) in this supplementary material.

**5. References：**

1. Li, W. *et al*. Dynamic analysis and rotation experiment of an optical-trapped microsphere in air. *Appl. Opt.* **57**, 823–828 (2018)
2. Neumann, E. -G. Gaussian beams in Single-Mode Fibers: Fundamentals (Springer, 1988), pp. 35–60.
3. Sidick E., Collins S. D., Knoesen A. Trapping forces in a multiple-beam fiber-optic trap. *Applied Optics*, **36**, 6423–33 (1997)..
4. N Grønbech-Jensen, N., Farago, O. A simple and effective Verlet-type algorithm for simulating Langevin dynamics. *Mol. Phys.* **111**, 983–991 (2013).
5. Li, T. C., Kheifets, S., Medellin, D., Raizen, M. G. Measurement of the instantaneous velocity of a Brownian particle. *Science* **328**, 1673–1675 (2010).
